# In Vivo Tracking of Chemokine Receptor CXCR4-Engineered Mesenchymal Stem Cell Migration by Optical Molecular Imaging

**DOI:** 10.1155/2017/8085637

**Published:** 2017-06-27

**Authors:** Senthilkumar Kalimuthu, Ji Min Oh, Prakash Gangadaran, Liya Zhu, Ho Won Lee, Ramya Lakshmi Rajendran, Se hwan Baek, Yong Hyun Jeon, Shin Young Jeong, Sang-Woo Lee, Jaetae Lee, Byeong-Cheol Ahn

**Affiliations:** Department of Nuclear Medicine, Kyungpook National University School of Medicine and Hospital, Daegu, Republic of Korea

## Abstract

CXCR4, the stromal cell-derived factor-1 receptor, plays an important role in the migration of hematopoietic progenitor/stem cells to injured and inflamed areas. Noninvasive cell tracking methods could be useful for monitoring cell fate. Therefore, in this study, we evaluated the efficacy of an intravenous infusion of genetically engineered mesenchymal stem cells (MSCs) overexpressing CXC chemokine receptor 4 (CXCR4) to home to the tumor, by optical imaging. We constructed a retroviral vector containing CXCR with dual reporter genes, *eGFP* and *Fluc2*, under the control of an EF1*α* promoter (pBABE-EF1*α*-CXCR4-eGFP-IRES-Fluc2). We also developed an eGFP-Fluc2 construct in the Retro-X retroviral vector (Retro-X-eGFP-Fluc2). MSCs were transduced with retroviruses to generate CXCR4-overexpressing MSCs (MSC-CXCR4/Fluc2) and MSCs (MSC/Fluc2). CXCR4 mRNA and protein expression was confirmed by RT-PCR and Western blotting, respectively, and it was higher in MSC-CXCR4/Fluc2 than in naive MSCs. eGFP expression was confirmed by confocal microscopy. The transfected MSC-CXCR4/Fluc2 cells showed higher migratory capacity than naive MSCs observed in Transwell migration assay. The in vivo migration of CXCR4-overexpressing MSCs to MDAMB231/Rluc tumor model by BLI imaging was also confirmed. Intravenous delivery of genetically modified MSCs overexpressing CXCR4 with a *Fluc2* reporter gene may be a useful, noninvasive BLI imaging tool for tracking cell fate.

## 1. Introduction

Worldwide, there are 1.38 million women with breast cancer [1]. Breast cancer is one of the most common cancers among women, especially in less developed countries and the second leading cause of cancer death [2]. Most patients with breast cancer eventually undergo chemotherapy. However, only a few patients treated with chemotherapy show long-term reduction, and response failures are common due to chemoresistant tumors [3]. Triple negative breast cancer (TNBC) is a subtype of tumors that do not clinically express human epidermal growth factor receptor 2 (HER-2), progesterone receptor (PR), or estrogen receptor (ER). This subtype, which is associated with poor prognosis, accounts for approximately 15–20% breast cancers. However, TNBC is accountable for a disparate number of deaths, and there is no effective, specific-targeted therapy available [4]. Mesenchymal stem cell- (MSC-) based homing studies may lead to a better approach for treating such breast cancers.

MSCs are self-renewing, multipotent, progenitor cells with multilineage potential that can differentiate into cells of mesodermal origin, such as adipocytes, osteocytes, and chondrocytes [5, 6]. MSC-mediated gene therapy has been investigated as an option for the treatment of various diseases, including articular cartilage damage, hemophilia, and myocardial infarction [7–11]. MSCs are most commonly isolated from bone marrow [12], although they are also isolated from other tissues, including adipose tissue [13, 14], the placenta [15], amniotic fluid [16], and umbilical cord blood [17, 18]. Owing to their accessibility and convenient expansion protocols, MSCs have been recognized as promising candidates for cellular therapy. Bone marrow is a rich source of MSCs; therefore, it is often used for isolating MSCs. MSCs can be expanded by ex vivo culture, thereby making them good vehicles for in vivo gene delivery.

MSCs have the ability to home to damaged tissue sites. When MSCs are systemically delivered or exogenously administered to humans and animals, they specifically migrate to inflammation sites [5], although many intravenously administered MSCs become trapped in the lungs [19, 20]. MSC homing to inflammation sites has been demonstrated, and numerous cell trafficking-related molecules are involved, mainly adhesion molecules, chemokines, and matrix metalloproteinases. Among these, the CXC motif chemokine ligand 12-CXC motif chemokine receptor 4 (CXCR4) and CC motif chemokine ligand 2-CC motif chemokine receptor 2 (CCR2) ligand-receptor pairs have been actively studied [21, 22].

Although CXCR4 is highly expressed by MSCs within the bone marrow [23], its expression is markedly reduced during ex vivo expansion [24], which decreases their ability to home to injured sites. Therefore, the activation of CXCR4 could make MSCs migrate to CXCR4 ligands. CXCR4, the stromal cell-derived factor-1 (SDF) receptor, plays an important role in the migration of hematopoietic progenitor/stem cells [25]. The overexpression of CXCR4 in human hematopoietic stem cells or CD34+ progenitors has been shown to improve chemotaxis, migration, and homing [26]. However, there are limited noninvasive studies showing the migration potency of these therapeutic CXCR4-overexpressing cells to cancer cells in animal models.

Recent developments in noninvasive imaging tools continue to reinforce the utility of molecular imaging for biological research. These imaging technologies, coupled with the development of cell-based therapies, are leading a transformation in cell tracking. The transplanted cells are visualized via molecular imaging tools, which shows the fate, function, migration, and homing of the cells, and such in vivo cell monitoring strategies are highly valuable for the development of cell-based therapies [27]. Moreover, cell tracking using bioluminescence imaging (BLI) offers the highest sensitivity in small animal experiments due to the absence of endogenous luciferase expression in mammalian cells [28]. Therefore, in the current study, we established CXCR4-overexpressing MSCs, containing a *Fluc2* reporter gene, and evaluated their in vivo migrating efficiency to triple negative breast cancer by BLI.

## 2. Materials and Methods

### 2.1. Chemicals

DMEM-high was obtained from Hyclone (Logan, UT, USA). Antibiotics were obtained from Gibco-Invitrogen (Carlsbad, CA, USA), and the anti-CXCR4 antibody (rabbit polyclonal) was obtained from Abcam (Cambridge, MA, USA). The CXCR4-eGFP vector was a kind gift from Dr. Peter L. Hordijk (University of Amsterdam, Netherlands). The pBABE vector was purchased from Addgene (Cambridge, MA, USA).

### 2.2. Cell Culture

Bone marrow-derived mouse MSCs were purchased from GIBCO (Invitrogen). Cells were grown in DMEM-high with 10% fetal bovine serum and 1% antibiotic-antimycotic (Gibco, Invitrogen) and maintained in a humidified incubator at 37°C with 5% CO_2_.

### 2.3. Retroviral Vector and MSC Transduction

To track transplanted cells in vivo, MSCs were transduced with the retroviral vector pBABE carrying an EF1*α* promoter for CXCR4 expression. To generate a triple gene construct, we inserted the CXCR4-eGFP cassette along with *Fluc2* by using linker molecules and internal ribosome entry sites (IRES). The designed construct was inserted into the pBABE vector after under the control of the EF1*α* promoter to generate the final construct, pBABE-EF1*α*-CXCR4-eGFP-IRES-Fluc2. We also generated another construct, Retro-X-eGFP-IRES-Fluc2 without CXCR4. The gene construct was shown in Additional File 1 available online at https://doi.org/10.1155/2017/8085637. Both plasmid retroviruses were produced in Gryphon E (Allele Biotechnology, San Diego, CA, USA) cells after transfection using the CaPO_4_ method. After 2 days, the medium was collected and filtered, and the filtrate was then transduced into MSCs. The transduced MSCs, with and without CXCR4 overexpression, were sorted to generate stable cell lines and called MSC-CXCR4/Fluc2 and MSC/Fluc2, respectively. The Fluc activity was measured by IVIS Lumina II by adding D-Luciferin 150 *μ*g/100 *μ*l final concentration.

### 2.4. Confocal Microscopy

Naive MSCs, MSC/Fluc2, and MSC-CXCR4/Fluc2 cells (5 × 10^5^) were plated in an 8-well cell chamber. Twenty-four hours after plating, the cells were washed twice with PBS, fixed with 4% paraformaldehyde, and washed three times with PBS. Finally, the slides were mounted with a coverslip using Vecta shield mounting medium containing DAPI (Vector Laboratories, Burlingame, CA, USA), and eGFP images were visualized by confocal laser microscopy (LSM 5 exciter; Zeiss, Germany).

### 2.5. Flow Cytometry

To confirm the identity of CXCR4-transduced MSCs, we assessed eGFP expression by flow cytometry. After stable transduction of MSC-CXCR4/Fluc2 cells, we compared the eGFP expression of MSC-CXCR4/Fluc2 cells to that of naive MSCs.

### 2.6. Lentiviral Transduction of Breast Cancer Cells

Triple negative breast cancer cells (MDAMB231) were transduced with lentiviral particles expressing mCherry-Rluc under the control of the CMV promoter (GeneCopoeia, Rockville, MD, USA). After transfection, cells were selected with puromycin to generate a stable cell line. The stable cell line was named MDAMB231/Rluc, and the cells were screened by optical imaging and fluorescence microscopy. For Rluc activity, substrate coelenterazine h (CTZ) (Perkin Elmer, Waltham, MA, USA) was added to each well, and Rluc activity was determined by BLI (Bioluminescence imaging).

### 2.7. RT-PCR

The transduced MSC-CXCR4/Fluc2 cell line was screened by RT-PCR. Total RNA was isolated from naive and transduced MSCs with TRIzol reagent (Invitrogen). The concentration and purity of the total RNA was determined by NanoDrop 2000 (Thermo Scientific, Wilmington, DE, USA), the ratio of which was 1.6–2.0. cDNA was synthesized from 1 *μ*g of total RNA using the TOPscript™ cDNA synthesis kit (Enzynomics, Daejeon, South Korea) according to the manufacturer's protocol. For CXCR4 gene amplification, glyceraldehyde-3-phosphate dehydrogenase (*GAPDH*) was used as a control for reverse transcription. The PCR reaction was performed with the i-Taq TM DNA Polymerase kit (iNtRON Biotechnology, Westchester, NY, USA) in an Eppendorf master cycler. The amplification protocol included an initial denaturation step at 95°C for 7 min and followed by 35 cycles of denaturation at 94°C for 40 s and extension at 60°C for 30 s, with a final extension at 72°C for 10 min. Amplification products were analyzed electrophoretically on a 1.0% agarose gel containing 0.5 mg of ethidium bromide (Bioneer, Alameda, CA, USA). The PCR bands were visualized with a Biodoc IT imaging system (UVP, Upland, CA, USA).

### 2.8. Immunoblot Analysis

Cell lysates containing equal amounts of protein (40 *μ*g) were separated by SDS-PAGE and electrophoretically transferred to a PVDF membrane. The PVDF membrane was blocked with 5% nonfat dry milk in PBS-Tween-20 (0.1%, *v*/*w*) for 2–4 hours. The membrane was incubated with an anti-CXCR4 antibody (rabbit polyclonal, ab2074-Abcam, 1 : 1000) overnight. Then, an HRP-conjugated anti-rabbit (Cell Signaling, Beverly, MA, USA) was used as the secondary antibody. Immunoreactive protein was visualized by chemiluminescence (Power OptiECL, Animal Genetics, South Korea) and quantified with Image Scanner. *β*-actin was used as an internal control.

### 2.9. Transwell Migration Assay

Migration assays were carried out in a 24-well Transwell using polycarbonate membranes with 8 μm pores (Corning Costar, Cambridge, MA, USA). MSC-CXCR4/Fluc2, at a density of 1 × 10^5^ cells in 500 *μ*l of serum-free medium, were placed in the upper chamber of the Transwell assembly. The lower chamber contained 0.5% FBS. After incubation at 37°C and 5% CO_2_ for 6 h, the upper surface of the membrane was scraped gently to remove nonmigrating cells and washed with PBS. Then, the migrated cells on the lower surface of the membrane were fixed in 4% paraformaldehyde for 15 minutes and stained with 0.1% crystal violet for 10 minutes. The number of migrating cells was photographed under a phase contrast microscope, and migrated cells were counted three different fields.

### 2.10. In Vivo MSC Migration to Tumors

BLI was used to assess the in vivo migration of transduced MSCs. All animal experiments were performed in accordance with institutional guidelines and were approved by the Institutional Animal Care and Use Committee of the Kyungpook National University of Korea. MDAMB231/Rluc breast cancer tumor xenografts were established in 6-week-old female nude mice by injection of 5 × 10^6^ cells into the right flank. Tumor production 1 month after tumor cell inoculation was assessed by measuring Rluc activity by BLI. Tumor-bearing mice received 1 × 10^6^ MSCs in PBS via IV injection, and then the migration of the transduced MSCs was tracked in vivo by BLI. We separated tumor-bearing mice into two groups, namely, MSC/Fluc2 and MSC-CXCR4/Fluc2. Each group consisting of three mice.

BLI was performed with an IVIS Lumina II optical imaging system (Caliper Life Sciences, Hopkinton, MA). For Fluc activity, five minutes prior to imaging, each mouse was given a 100 *μ*l of D-luciferin (150 mg/kg body weight) by IP injection. The luminescent photographic images of each mouse were acquired with one-minute exposure time and medium binning. For Rluc activity, coelenterazine 20 *μ*l (10 mg/5 ml ethanol) was mixed with sodium phosphate buffer and injected 200 *μ*l per mouse by IV and immediately started the IVIS imaging with less than 20 seconds exposure time and medium binning for measurements up to maximum photon flux. The images were quantified separately drawn a ROI at lungs and tumor region and then measured by live image software.

### 2.11. Immunohistochemistry

To confirm the MSC/Fluc2 and MSC-CXCR4/Fluc2 after 24 h tracking, tissues fixed in 10% formalin were dehydrated and embedded in paraffin. Paraffin-embedded sections of 5 *μ*m were prepared according to standard protocols. Anti-GFP antibody (Millipore, USA) was added and stained with a DAB kit. The GFP-positive staining was visualized under a light microscope at 40x magnifications.

## 3. Statistical Analysis

In vitro experiments were performed in three individual measurements, and in vivo experiments with three mice data were used for analyzing the means ± standard deviation (SD); the *P* value <0.05 was considered as statistically significant by Student *t*-test. *R*^2^ was analyzed by using the Excel sheet.

## 4. Results

### 4.1. Characterization of CXCR4-Transduced MSCs

**Figure 1:**
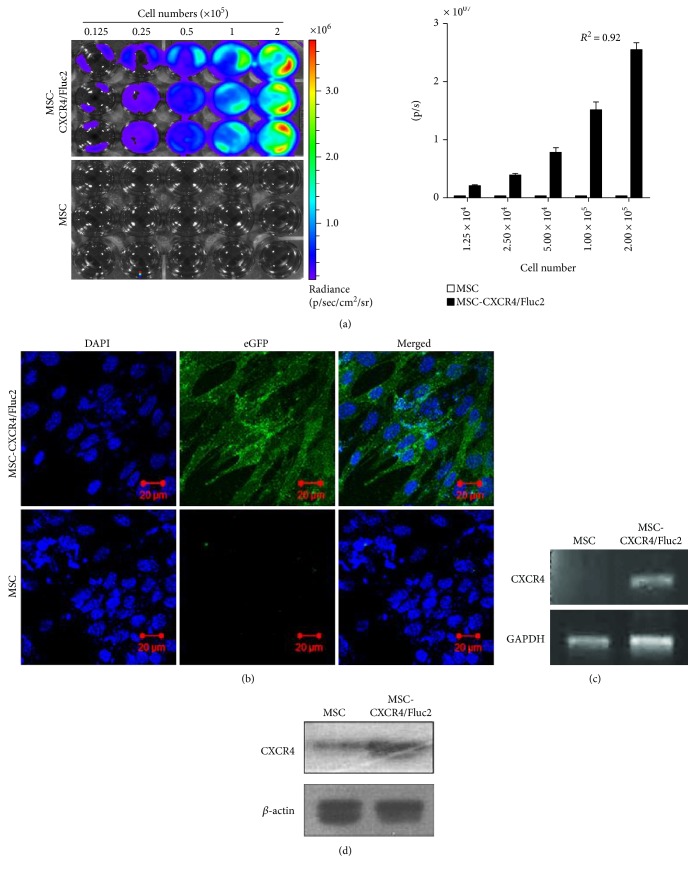
Transduction of CXCR4 in mesenchymal stromal cells (MSCs) with reporter genes. (a) Fluc activity and quantitative bioluminescent imaging (BLI) data of CXCR4-transduced MSCs with different cell numbers. (b) Enhanced green fluorescent protein (eGFP) expression analysis by confocal microscopy imaging and (c) CXCR4 mRNA expression analysis by RT-PCR. (d) Protein expression of CXCR4 by Western blot analysis.

Transduced MSC-CXCR4/Fluc2 cells were generated by retroviral transfection, and eGFP-positive cells were sorted by FACS Aria III. The BLI signal in MSC-CXCR4/Fluc2 cells increased as the number of cells increased (Figure 1(a), *R*^2^ = 0.92), and eGFP was assessed by confocal microscopy (Figure 1(b)). We also examined eGFP expression by flow cytometry (Additional Figure 2). CXCR4 mRNA and protein expression were confirmed by RT-PCR and Western blotting, respectively (Figures 1(c) and 1(d)).

### 4.2. Characterization of MSC/Fluc2

**Figure 2:**
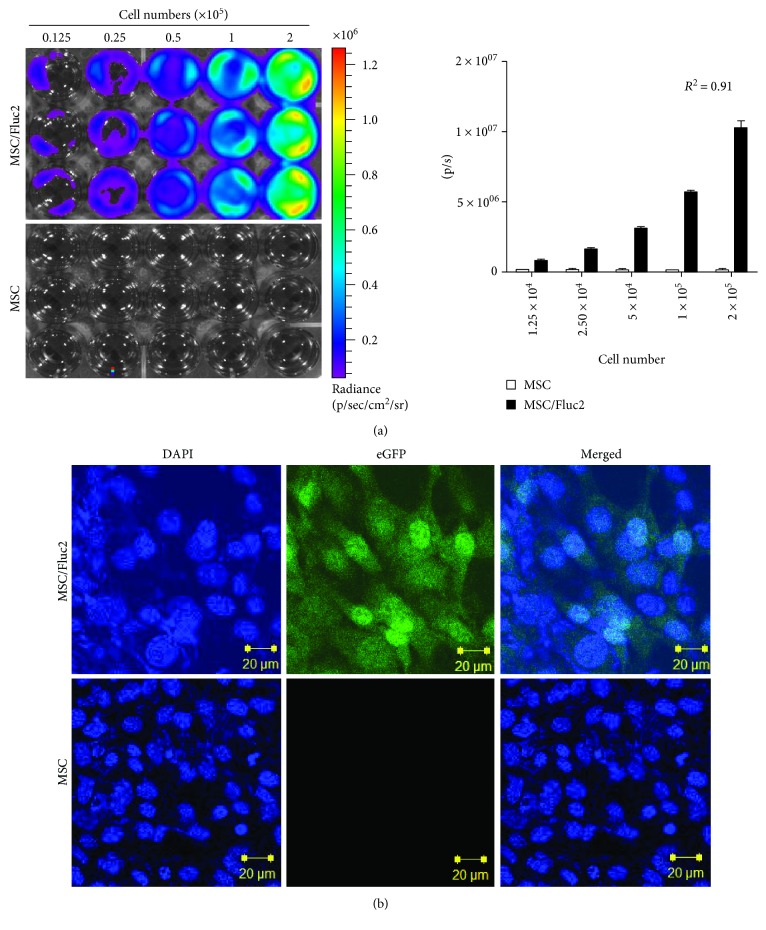
Characterization of MSC/Fluc2 cells (a) BLI and quantitation of Fluc activity in transduced MSCs (MSC/Fluc2) at various concentrations. (b) eGFP confocal microscopy in transduced MSCs (MSC/Fluc2).

The eGFP- and Fluc2-transduced MSC/Fluc2 cell lines were created by retroviral transfection, and then eGFP-positive cells were sorted by FACS Aria III. The BLI signal in MSC/Fluc2 increased as the number of cells increased (Figure 2(a), *R*^2^ = 0.91); eGFP expression was shown by fluorescent microscopy (Figure 2(b)).

### 4.3. Characterization of MDAMB231/Rluc Cells

**Figure 3:**
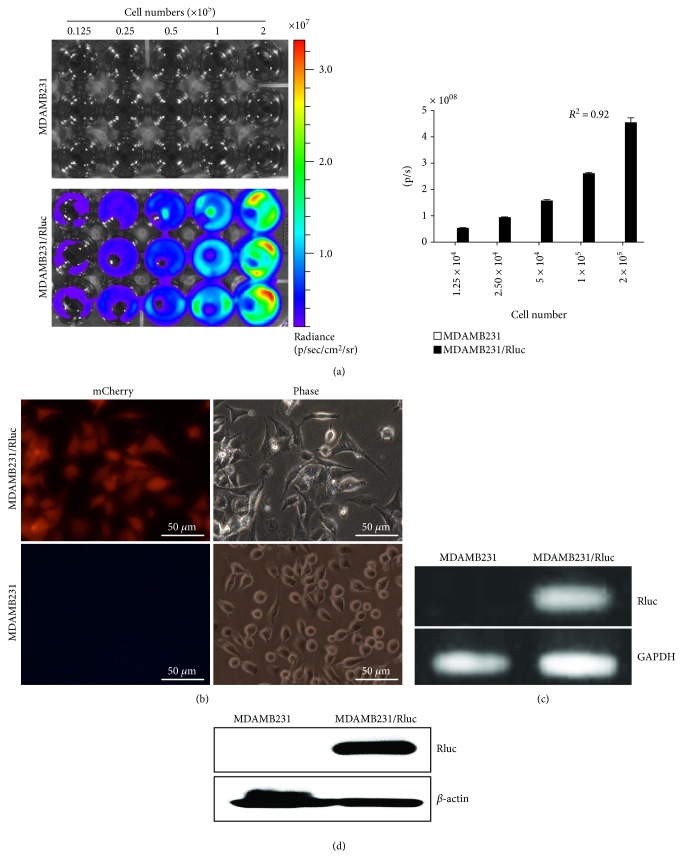
Characterization of MDAMB231/Rluc. (a) Rluc activity assessed by bioluminescent imaging (BLI) and quantitative analysis of MDAMB231/Rluc at different concentrations. (b) Transduced MDAMB231/Rluc cells were strongly positive for mCherry by fluorescence microscopy. (c) Rluc mRNA expression by RT-PCR. (d) Rluc protein expression by Western blotting.

The Rluc activities of stably transduced MDAMB231/Rluc and parental MDAMB231 cells were determined by BLI. The bioluminescence activity of MDAMB231/Rluc cells increased as the number of cells increased (Figure 3(a); *R*^2^ = 0.92). mCherry was confirmed by fluorescent microscopy (Figure 3(b)). Rluc mRNA and protein expression were confirmed by RT-PCR and Western blotting, respectively (Figures 3(c) and 3(d)).

### 4.4. In Vitro Migration of Transduced MSCs

**Figure 4:**
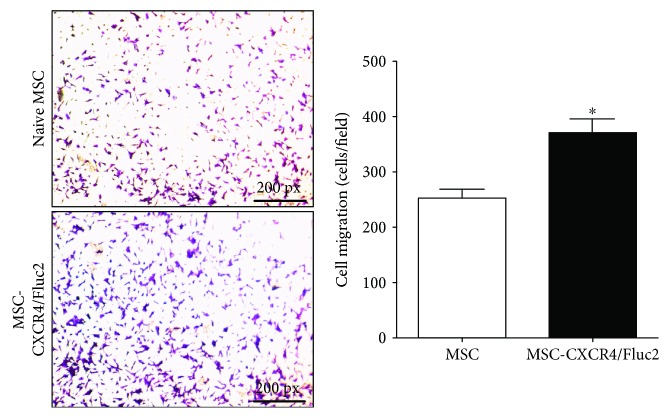
Chemotaxis assay for MSC-CXCR4/Fluc2. MSCs and MSC-CXCR4/Fluc2 in serum-free medium were placed into the upper well of a 24-well Transwell plate, and 0.5% FBS was added to the lower well. The plates were incubated for 6 h, and then cells that migrated into the lower well were fixed, stained with crystal violet, and photographed in 4x magnification. One representative experiment of three is shown.

In an in vitro study, bone marrow-derived MSCs containing CXCR4 (MSC-CXCR4/Fluc2) showed more migration into the lower compartment (containing 0.5% FBS) than naive MSCs in a chamber assay (Figure 4) after 6 hours.

### 4.5. In Vivo Migration of MSCs

**Figure 5:**
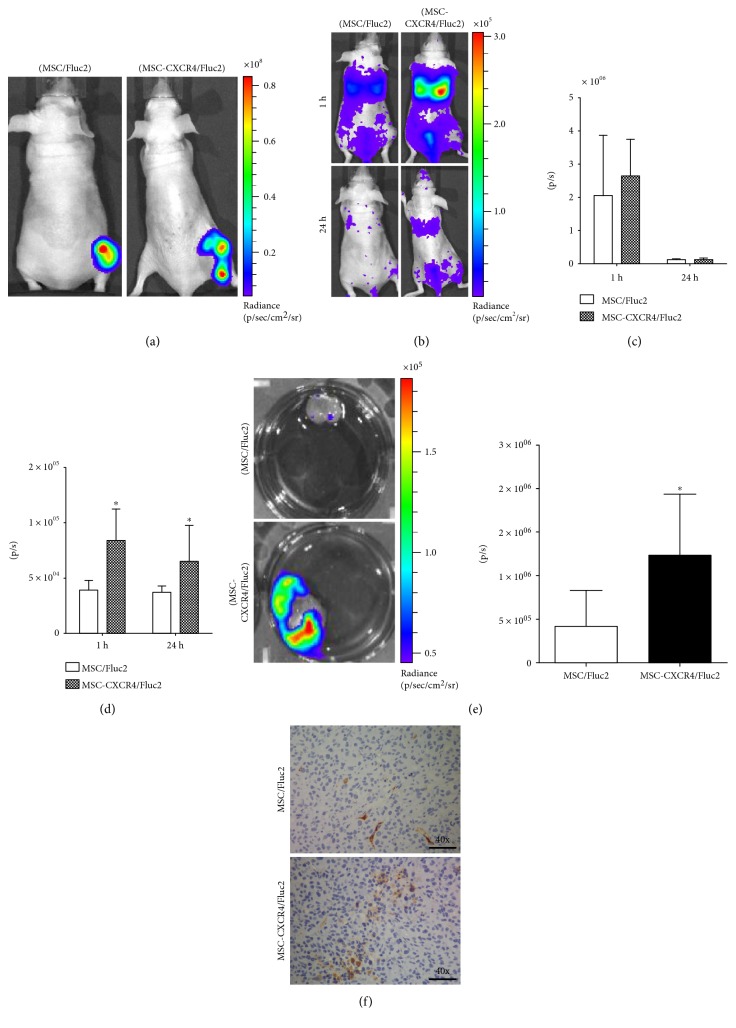
In vivo migration and localization of transplanted MSC-CXCR4/Fluc2 in the tumors. (a) Rluc activity of MDAMB231/Rluc tumor xenografts. (b) Fluc activity of migrated MSC-CXCR4/Fluc2 and MSC/Fluc2 in breast cancer xenografts. (c) Quantitative data of Fluc activity from the lungs. (d) Quantitative data of Fluc activity from tumor. (e) Ex vivo Fluc imaging of migrated MSC-CXCR4/Fluc2 and MSC/Fluc2. (f) Immunohistochemistry analysis of GFP for MSC/Fluc2 and MSC-CXCR4/Fluc cells in MDAM231/Rluc tumor. Data were analyzed from 3 mice with the means ± standard deviation (SD), and the *P* value <0.05 was considered as statistically significant by Student *t*-test.

To monitor the in vivo migration of systemically transduced MSCs, we performed BLI to track the MSCs. In this study, we confirmed that the migration of MSCs toward breast cancer cells after intravenous delivery could be visualized by in vivo cell tracking using BLI. We used noninvasive systemic delivery and in vivo imaging to examine the tumor-targeting property of transduced MSCs. MSC/Fluc2 and MSC-CXCR4/Fluc2 were injected (1 × 10^6^) into tumor-bearing mice through the tail vein, and MSC localization was assessed by BLI at 1 and 24 hours after injection (Figure 5(b)). The imaging showed that at 1 and 24 h after tail vein injection, the MSCs moved from the lung to the tumor sites. We quantified the lung and tumor region Fluc activity by drawn ROI separately at both regions and found that Fluc activity was decreased at 24 h (Figure 5(c)) in the lungs. More MSC-CXCR4/Fluc2 migrated to tumor sites than MSC/Fluc2 at both 1 and 24 hours after tail vein injection (Figure 5(d)). We also confirmed higher Fluc activity of the MSCs in the tumors by ex vivo tumor imaging (Figure 5(e)). Furthermore, MSC/Fluc2 and MSC-CXCR4/Fluc2 cells after 24 h injection in tumor regions, we performed IHC for GFP-positive cells. IHC results showed that the positive staining of MSC-CXCR4/Fluc2 was higher, when compared, than MSC/Fluc2 (Figure 5(f)). The Rluc activity of MDAMB23/Rluc was shown in a xenograft mice model (Figure 5(a)), and the in vivo results showed that the tumor recruits more CXCR4-overexpressing MSCs than native MSCs.

## 5. Discussion

For our study, MSCs were labeled with luciferase by retroviral transduction, and in vitro and in vivo migration studies demonstrated that engineered MSCs showed enhanced migratory ability towards tumors. These Fluc2-labeled MSCs could be used for noninvasive in vivo visualization and have potential for use in preclinical protocols. The increasing clinical use of MSCs in cellular and gene therapies for various diseases, including genetic and malignant disorders, is limited by the low and/or transient engraftment of transplanted MSCs [29]. The main source of MSCs, bone marrow, was taken into consideration for clinical use. The transplanted MSCs were engineered to contain therapeutic genes that display tropism for the tumors, and MSCs have been shown to exert significant antitumor effects in a human glioma xenograft mouse model [30–32]. The progenitor cells express CXCR4, which helps target the cells to injury sites when bound to SDF-1. Cultured bone marrow-derived MSCs are strongly attracted to SDF-1 due to the expression of CXCR4 [33]. However, during expansion of the MSCs in ex vivo culture, CXCR4 expression is downregulated; thus, the MSCs lose their migration potential [21]. Therefore, CXCR4 overexpression was used to increase the homing of systemically delivered MSCs towards infarcted brain. The overexpression of CXCR4 mediated the migration of MSCs towards an SDF-1 gradient in an in vitro migration assay, and systemically infused CXCR4-engineered MSCs showed strong migration towards infarcted brain when compared to naive MSCs. The CXCR4 modification did not influence the proliferation or phenotype of MSCs [34]. Genetically modified MSCs expressing CXCR4 have a typical fibroblast-like structure, similar to that of naive MSCs, and their viability and proliferation were not changed after 6 days of culture [10]. Here, we successfully transduced MSCs with CXCR4 and reporter genes (*eGFP* and *Fluc2*), and we confirmed Fluc2 activity by BLI and CXCR4 mRNA and protein expression (Figure 1). We also confirmed the in vitro migration of MSCs and MSC-CXCR4/Fluc2, as 0.5% FBS-dependent migration in a Transwell migration assay, which demonstrated the chemotactic activity of MSC-CXCR4/Fluc2 (Figure 4). In the current study, optical molecular imaging revealed that CXCR4-overexpressing MSCs migrated to the MDAMB231/Rluc xenografts. CXCR4-overexpressing MSCs could be useful for SDF1*α*-secreting breast cancer therapeutic drug delivery.

MSCs express numerous receptors and cell adhesion molecules that are involved in migration and homing to certain diseased tissues, and a significant portion are dependent on CXCR4-mediated homing [35, 36]. The gene expression profiles of MSCs exposed to various tumor cell-derived conditioned mediums revealed the downregulation of matrix metalloproteinase-2 and the upregulation of CXCR4. This suggests that CXCR4 and MMP-2 are involved in the multistep migration of MSC [33]. However, in previous studies, the migration of labeled MSCs was confirmed in tissue sections by immunohistochemistry, fluorescent methods, or PCR, only after the animal had been sacrificed. These methods require animals to be sacrificed at multiple time points and preparation and analysis of tissues. Because of these limitations, we used noninvasive in vivo live imaging to visualize and track the cells to assess the migration and distribution of engineered MSCs over a range of time points in the same animal.

In the current study, we found that systemically injected MSCs were distributed to the tumor region in breast cancer (MDAMB231/Rluc) xenograft mice. The injected MSCs first will go the lungs, then some time later, they will migrate to various parts of the body. After 1 hour, we found that the transduced MSCs, with and without CXCR4, reached the tumor site and remained, even after 24 hours (Figure 5(b)). The decrease of MSC/Fluc cells in the lung after 24 h due to the migration of cells to other inner parts of the body may be the reason. The exact mechanism for MSC-CXCR4/Fluc2 long time in the lungs is unclear. The Fluc signal from the spine region was due to cells may be present in the bone marrow of the spine region. Transplanted MSC homing to bone marrow [37]. We also confirmed the Fluc2 activity of MSC/Fluc2 and MSC-CXCR4/Fluc2 by ex vivo BLI imaging (Figure 5(e)), and GFP-positive cells by IHC staining showed that higher stain was obtained from MSC-CXCR4/Fluc2 injected cells (Figure 5(f)). Systemically delivered MSCs preferentially target sites of ischemic lesions, injuries, and tumors in the brain, despite their predominant entrapment in the lung vasculature [20, 38]. In the present study, we found that the MSCs were transiently trapped in the lungs but then migrated over time to the tumor region, which means that MSCs administered systemically via the tail vein can reach tumors, even though some cells remain in the lungs. In addition, more CXCR4-overexpressing MSCs accumulated in the tumor than non-CXCR4-overexpressing MSCs. This might be due to the release of chemotactic factors from the tumors that attract the CXCR4-overexpressing MSCs.

## 6. Conclusion

In the current study, we showed that optical imaging can be used to reveal the distribution and homing of systemically injected MSC-CXCR4/Fluc2 and MSC/Fluc2 toward tumor sites in mice bearing breast cancer xenografts. This suggests that CXCR4-mediated cell-based therapeutics can be used to treat breast cancer. Optical imaging-based in vivo cell tracking might be a prospective imaging technique to develop MSC-mediated therapies.

## Supplementary Material

Additional file 1. Schematic diagram of vector construct . (A) Retroviral vector for double reporter gene for making MSC/Fluc2. (B) Retroviral vector of CXCR4 containing double reporter gene for making MSC-CXCR4/Fluc2. (C) Lentiviral particles for double reporter gene containing Rluc and mCherry for MDAMB-231/Rluc. Additional file 2. eGFP analysis by FACS of MSC/CXCR4-Fluc2 cells. The gating strategy included two successive gates: a first gate on SSC-H and FSC-H to select cells (P1), and for the histogram another gate (P2) drawn for confirm the GFP positive cells. The experiments were performed in triplicate used for analyzing the means ± standard deviation (SD).
