# Corrigendum to “In Vivo Tracking of Chemokine Receptor CXCR4-Engineered Mesenchymal Stem Cell Migration by Optical Molecular Imaging”

**DOI:** 10.1155/2020/8275897

**Published:** 2020-11-28

**Authors:** Senthilkumar Kalimuthu, Ji Min Oh, Prakash Gangadaran, Liya Zhu, Ho Won Lee, Ramya Lakshmi Rajendran, Se hwan Baek, Yong Hyun Jeon, Shin Young Jeong, Sang-Woo Lee, Jaetae Lee, Byeong-Cheol Ahn

**Affiliations:** Department of Nuclear Medicine, Kyungpook National University School of Medicine and Hospital, Daegu, Republic of Korea

In the article titled “In Vivo Tracking of Chemokine Receptor CXCR4-Engineered Mesenchymal Stem Cell Migration by Optical Molecular Imaging” [1], it was found that the image plates in Figures 1(a), 2(a), and 3(a) were incorrect. The errors are mainly in the published MSC plate images of Figures 1(a) and 2(a) and the MDA-MB-231 plate image of Figure 3(a). The authors confirmed that these corrections do not affect the results and conclusion of the article. The corrected versions of Figures 1–3 are shown below.

## Figures and Tables

**Figure 1:**
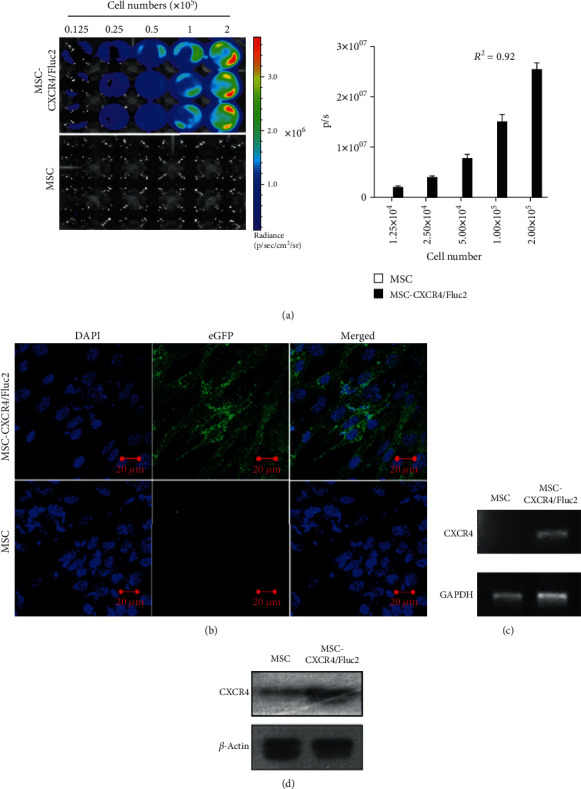
Transduction of CXCR4 in mesenchymal stromal cells (MSCs) with reporter genes. (a) Fluc activity and quantitative bioluminescent imaging (BLI) data of CXCR4-transduced MSCs with different cell numbers. (b) Enhanced green fluorescent protein (eGFP) expression analysis by confocal microscopy imaging and (c) CXCR4 mRNA expression analysis by RT-PCR. (d) Protein expression of CXCR4 by Western blot analysis.

**Figure 2:**
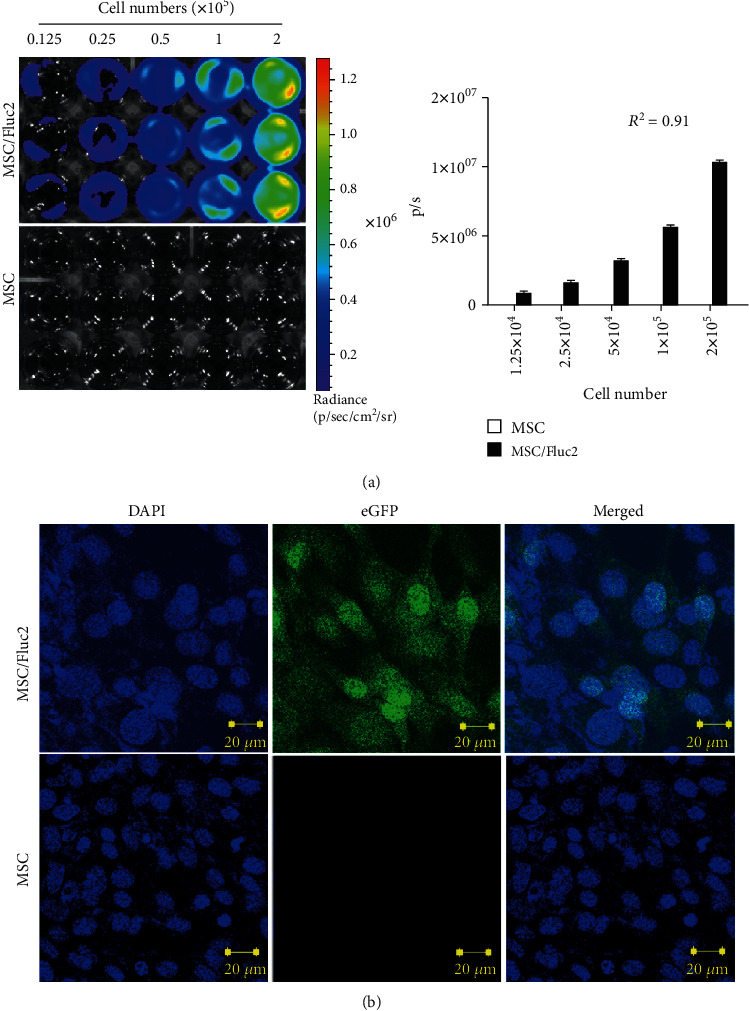
Characterization of MSC/Fluc2 cells (a) BLI and quantitation of Fluc activity in transduced MSCs (MSC/Fluc2) at various concentrations. (b) eGFP confocal microscopy in transduced MSCs (MSC/Fluc2).

**Figure 3:**
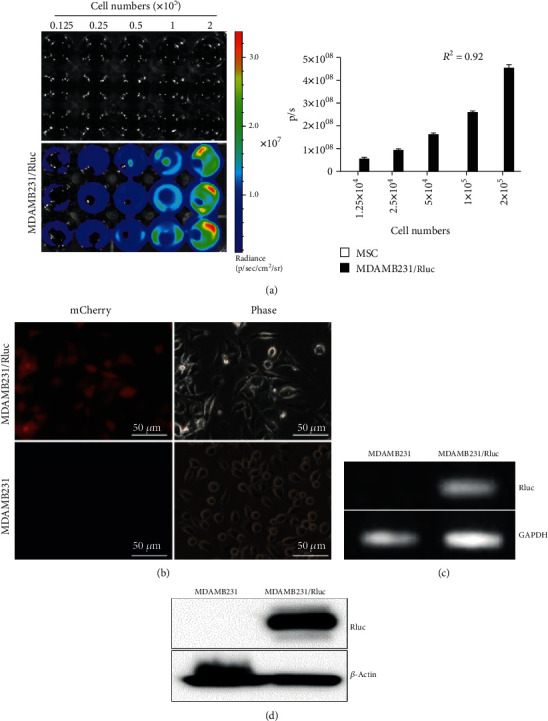
Characterization of MDAMB231/Rluc. (a) Rluc activity assessed by bioluminescent imaging (BLI) and quantitative analysis of MDAMB231/Rluc at different concentrations. (b) Transduced MDAMB231/Rluc cells were strongly positive for mCherry by fluorescence microscopy. (c) Rluc mRNA expression by RT-PCR. (d) Rluc protein expression by Western blotting.
