# Supplementary material for: In Vivo Tracking of Chemokine Receptor CXCR4-Engineered Mesenchymal Stem Cell Migration by Optical Molecular Imaging
Source: Stem Cells Int. 2017 Jun 27;2017:8085637. doi: 10.1155/2017/8085637 (PMC5505027; doi:10.1155/2017/8085637)
Supplement: Supplementary file 1 — Additional file 1. Schematic diagram of vector construct . (A) Retroviral vector for double reporter gene for making MSC/Fluc2. (B) Retroviral vector of CXCR4 containing double reporter gene for making MSC-CXCR4/Fluc2. (C) Lentiviral particles for double reporter gene containing Rluc and mCherry for MDAMB-231/Rluc. Additional file 2. eGFP analysis by FACS of MSC/CXCR4-Fluc2 cells. The gating strategy included two successive gates: a first gate on SSC-H and FSC-H to select cells (P1), and for the histogram another gate (P2) drawn for confirm the GFP positive cells. The experiments were performed in triplicate used for analyzing the means ± standard deviation (SD). [file 8085637.f1.pptx]

## Slide 1
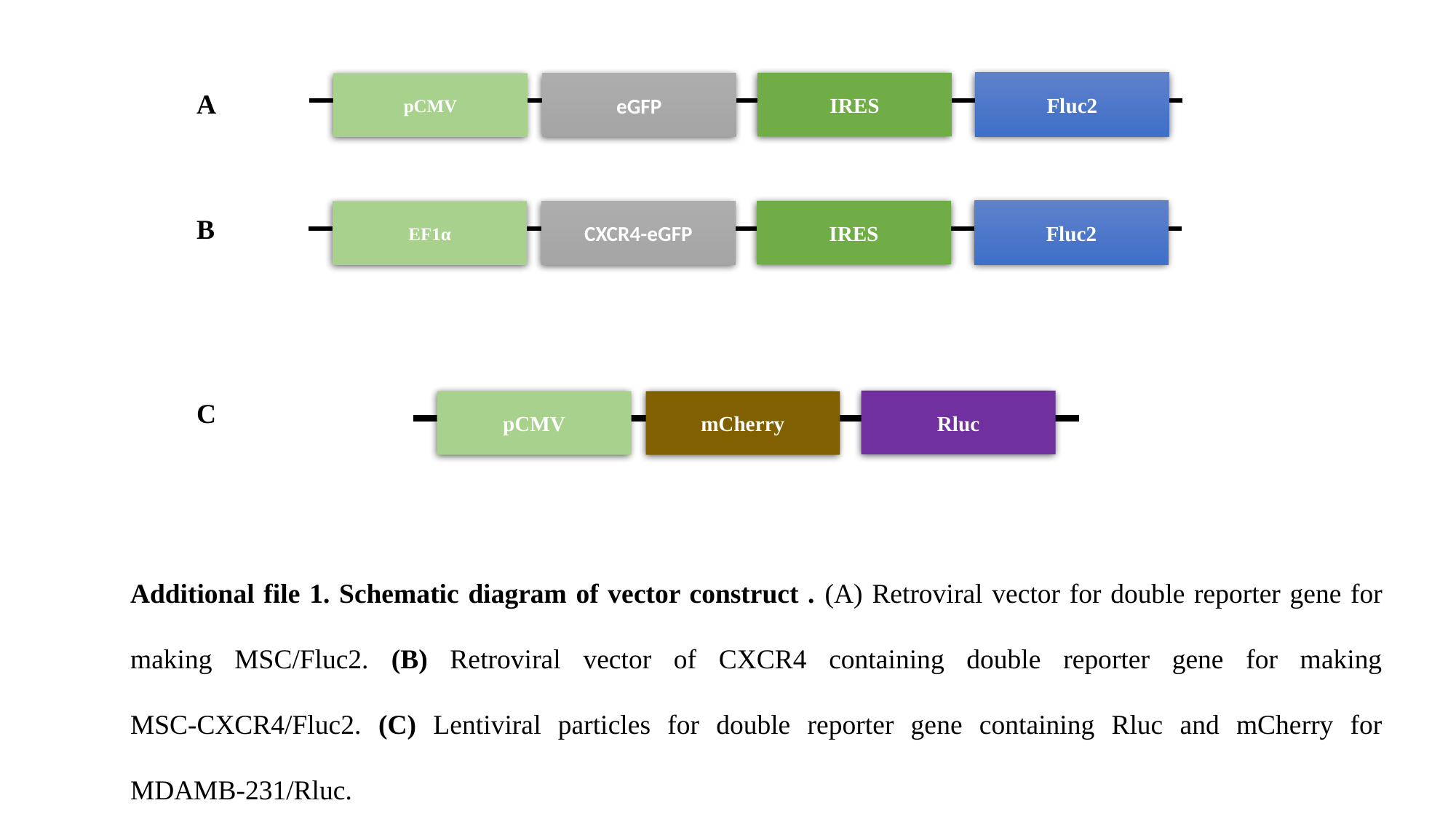

IRES
Fluc2
pCMV
eGFP
A
IRES
Fluc2
EF1α
CXCR4-eGFP
B
C
Rluc
pCMV
mCherry
Additional file 1. Schematic diagram of vector construct . (A) Retroviral vector for double reporter gene for making MSC/Fluc2. (B) Retroviral vector of CXCR4 containing double reporter gene for making MSC-CXCR4/Fluc2. (C) Lentiviral particles for double reporter gene containing Rluc and mCherry for MDAMB-231/Rluc.

## Slide 2
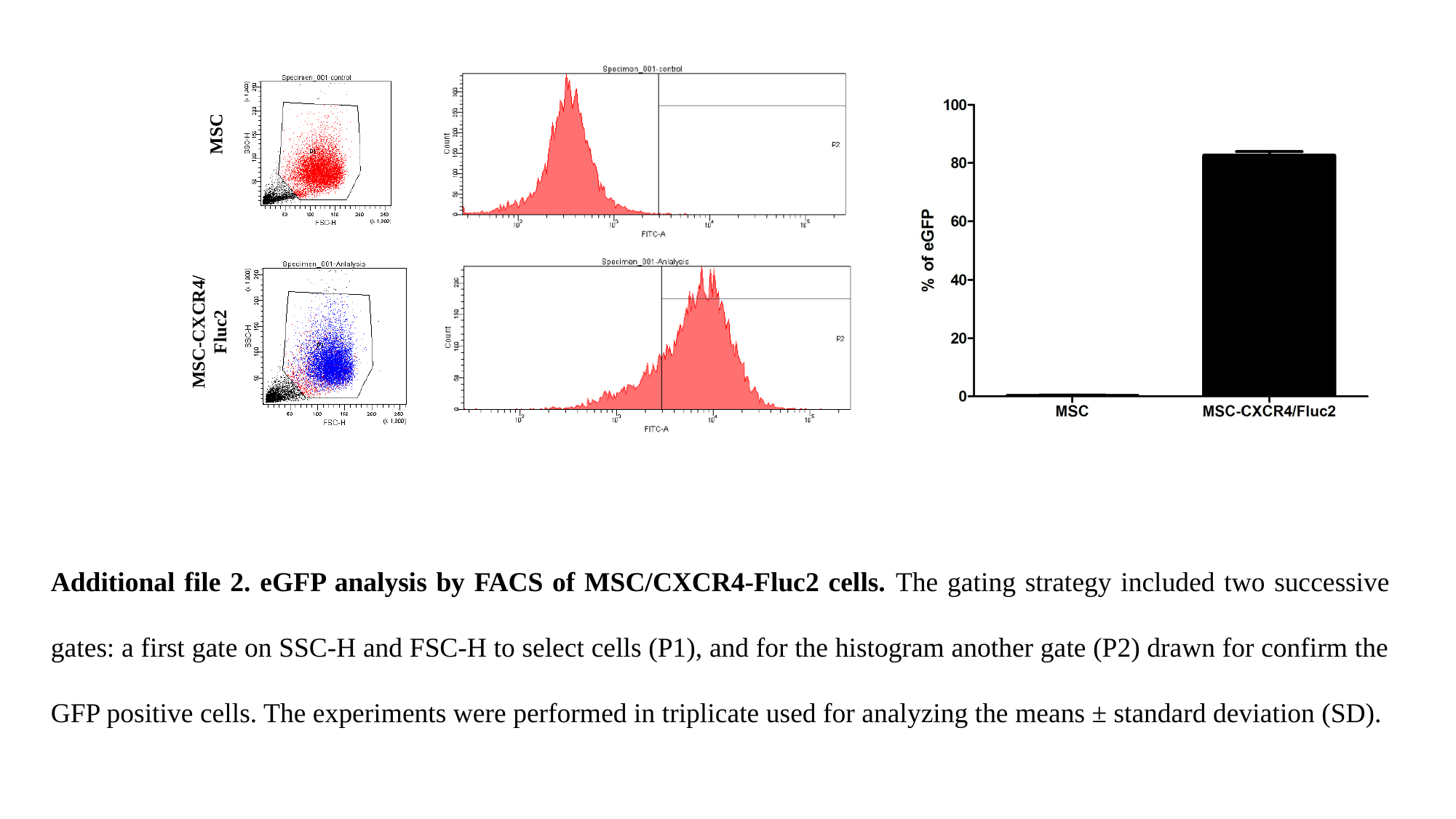

MSC
MSC-CXCR4/Fluc2
Additional file 2. eGFP analysis by FACS of MSC/CXCR4-Fluc2 cells. The gating strategy included two successive gates: a first gate on SSC-H and FSC-H to select cells (P1), and for the histogram another gate (P2) drawn for confirm the GFP positive cells. The experiments were performed in triplicate used for analyzing the means ± standard deviation (SD).
